# Carbon Dot Nanoparticles Exert Inhibitory Effects on Human Platelets and Reduce Mortality in Mice with Acute Pulmonary Thromboembolism

**DOI:** 10.3390/nano10071254

**Published:** 2020-06-28

**Authors:** Tzu-Yin Lee, Thanasekaran Jayakumar, Pounraj Thanasekaran, King-Chuen Lin, Hui-Min Chen, Pitchaimani Veerakumar, Joen-Rong Sheu

**Affiliations:** 1 Graduate Institute of Medical Sciences, College of Medicine, Taipei Medical University, Taipei 110, Taiwan; d119103001@tmu.edu.tw (T.-Y.L.); tjaya_2002@yahoo.co.in (T.J.); 2 Department of Chemistry, Fu Jen Catholic University, New Taipei City 242, Taiwan; ptsekaran@gmail.com; 3 Department of Chemistry, National Taiwan University, Taipei 10617, Taiwan; kclin@ntu.edu.tw; 4 Institute of Atomic and Molecular Sciences, Academia Sinica, Taipei 10617, Taiwan; 5 Department of Anatomy and Cell Biology, School of Medicine, College of Medicine, Taipei Medical University, Taipei 110, Taiwan; chm7805@tmu.edu.tw; 6 School of Chemistry, Madhurai Kamaraj University, Madhurai 625021, India

**Keywords:** nanoparticles, carbon dots, platelet aggregation, arterial thrombosis, signaling molecules, bleeding disorder

## Abstract

The inhibition of platelet activation is considered a potential therapeutic strategy for the treatment of arterial thrombotic diseases; therefore, maintaining platelets in their inactive state has garnered much attention. In recent years, nanoparticles have emerged as important players in modern medicine, but potential interactions between them and platelets remain to be extensively investigated. Herein, we synthesized a new type of carbon dot (CDOT) nanoparticle and investigated its potential as a new antiplatelet agent. This nanoparticle exerted a potent inhibitory effect in collagen-stimulated human platelet aggregation. Further, it did not induce cytotoxic effects, as evidenced in a lactate dehydrogenase assay, and inhibited collagen-activated protein kinase C (PKC) activation and Akt (protein kinase B), c-Jun N-terminal kinase (JNK), and p38 mitogen-activated protein kinase (MAPK) phosphorylation. The bleeding time, a major side-effect of using antiplatelet agents, was unaffected in CDOT-treated mice. Moreover, our CDOT could reduce mortality in mice with ADP-induced acute pulmonary thromboembolism. Overall, CDOT is effective against platelet activation in vitro via reduction of the phospholipase C/PKC cascade, consequently suppressing the activation of MAPK. Accordingly, this study affords the validation that CDOT has the potential to serve as a therapeutic agent for the treatment of arterial thromboembolic disorders

## 1. Introduction

Platelet activation has been associated with several thrombotic diseases. While it plays a vital role in regulating hemorrhagic events, hyperactivity can lead to a range of complications. In general, patients with cardio- and cerebrovascular ailments are found to have more reactive platelets than normal, healthy individuals. Thrombotic diseases pose a severe threat to humans as they may elicit significant injury and even lead to death. Several studies have recommended that intravenous heparin and tissue plasminogen activators are effective for treatment [1,2]; nevertheless, these are unsafe and may lead to severe bleeding and problems associated with reocclusion and reinfarction [3]. The inhibitors of antiplatelet drugs, such as the P_2_Y_12_ receptor, integrin α_IIb_β_3_, cyclooxygenase, and phosphodiesterase, are also widely used; however, they have serious limitations. Further, phosphatidylinositol 3-kinase inhibitors have been proposed as potential antithrombotic agents [4], but they also are associated with some major restrictions for use as drugs.

Nanoparticles can be defined as any particulate materials that range from 1 to 100 nm in size in at least one dimension [5], and they are ubiquitously distributed in the environment. In fact, humans are often exposed to airborne nanoparticles [6]. Their size can be manipulated to facilitate their passage across biological membranes and affect cell physiology [7]. Berry et al. [8] reported the substantial accumulation of nanoparticles in platelets in pulmonary capillaries and anticipated that there might be a predisposing factor for platelet aggregation and microthrombi formation. Nanoparticles are not proposed for systemic use as they can interfere with platelet function and increase the risk of cardiovascular diseases and vascular thrombosis [9]. However, some types of nanoparticles have been developed for therapeutic purposes that can target the injured vascular site to mimic platelet function [10] or enhance blood clotting [11]. Nevertheless, their undesirable, antiaggregating properties are of a significant concern in nanomedicine, impeding their widespread application in the clinical setting.

Carbon dots (CDOTs) have become the most important type of nanoparticles, considering their favorable biological properties. They are obtained from natural carbon sources and their average diameter is < 10 nm [12]. These nanoparticles have even been acquired from organic substances and are constant in water media, which is tremendously noteworthy from a biological point of view [13], especially in drug delivery, bioimaging, optical imaging, and biosensing due to their high biocompatibility [14]. A study reported that, in comparison to CDOTs, the application of noncarbon quantum dots has not received adequate attention, as they are associated with severe health and environmental concerns [15]. Yan et al. [16] reported the antihemorrhagic effects of novel water-soluble carbon quantum dots, and their results indicated the explicit hemostatic effect of these nanoparticles. CDOTs, isolated from egg yolk oil, demonstrated a hemostatic effect in mice via the stimulating intrinsic blood coagulation and fibrinogen systems [17]. Another relevant study showed that CDOTs from the Phellodendri Cortex carbonisatus considerably reduced bleeding time and coagulation parameters and significantly increased platelets without inducing toxicity when administered in mice [18]. Mariangela Fedel has recently reviewed the hemocompatibility of carbon nanostructures [19]. However, in general, the antiplatelet aggregating effects of CDOTs have not been extensively explored. Therefore, in this study, we investigated the antiplatelet and antithrombotic effects of a new type of CDOT in human platelets and mice, respectively.

## 2. Materials and Methods

### 2.1. Reagents

Collagen (type I), 9, 11-dideoxy-11α, 9α-epoxymethanoprostaglandin (U46619), and thrombin were purchased from Chrono-Log Corporation (Havertown, PA, USA). Anti-phospho-p38 mitogen-activated protein kinase (MAPK) (Thr180/Tyr182), anti-phospho-c-Jun N-terminal kinase (JNK) (Thr183/Tyr185), anti-phospho-(Ser) protein kinase C (PKC) substrate, anti-JNK polyclonal antibodies (pAb), and anti-p38 MAPK and anti-Akt monoclonal antibodies (mAb) were purchased from Cell Signaling Technology (Beverly, MA, USA). Anti-phospho-Akt (Ser473) pAb was purchased from Biorbyt (Cambridge, UK), and anti-pleckstrin (p47) pAb was purchased from Gene Tex (Irvine, CA, USA). Hybond-P polyvinylidene difluoride (PVDF) membranes, enhanced chemiluminescence (ECL) Western blotting detection reagent, and the analysis system were purchased from GE Healthcare Life Sciences (Buckinghamshire, UK). Horseradish peroxidase-conjugated goat anti-rabbit and anti-mouse immunoglobulin G antibodies were obtained from Jackson ImmunoResearch Laboratories (West Grove, PA, USA).

### 2.2. Preparation of CDOTs

Fresh garlic (*Allium sativum*) cloves were purchased from a local market in Taiwan, which were then peeled, crushed, and suspended in ultrapure water. This suspension was vigorously stirred for 1 h at 40 °C. The extract was filtered twice to remove insoluble materials and then freeze-dried. The obtained powder was stored at −20 °C until required. For CDOT synthesis, 100 mg of the garlic extract powder was dissolved in 3 mL water and poly (diallyldimethylammonium chloride) mixture (1/0.5, *v*/*v*). The clear transparent solution obtained was heated in a domestic microwave oven for 5 min at 600 W and then cooled to ambient temperature (25 °C). The obtained yellow-brown solution was diluted with ultrapure water and dialyzed against water for 2–3 h through a dialysis membrane.

### 2.3. Characterization of the Synthesized CDOTs

Crystallographic information pertaining to the CDOTs was collected using an analytical X-ray diffractometer (X’Pert PRO, Malvern, Worcestershire, UK) using Cu Kα radiation (λ = 0.1541 nm). A Fourier transform infrared (FT-IR) spectrometer (Bruker IFS28, Billerica, MA, USA) in the range of 4000–400 cm^−1^ was used for the characterization of functional groups on the surface of the CDOTs, with an average of 21 scans. The sample was prepared as pellets using KBr (IR grade). Ultraviolet–visible (UV–vis) spectra were documented using a Thermo Scientific Evolution 220 spectrophotometer (Waltham, MA, USA), whereas fluorescence spectral measurements were taken using a PerkinElmer LS-45 spectrometer (Waltham, MA, USA). The morphological information on the prepared CDOTs was obtained through field-emission transmission electron microscopy (FE-TEM, JEOL JEM-2100F, Akishima, Tokyo, Japan).

### 2.4. Preparation of Washed Human Platelets and Lactate Dehydrogenase (LDH) Release Assay

This study was performed in accordance with the Declaration of Helsinki, and the Institutional Review Board of Taipei Medical University approved all protocols (IRB: N201612050). All volunteers provided informed consent before they participated in this study. Anticoagulated human blood with acid–citrate–dextrose (1:9) was collected from healthy human volunteers who had not eaten any drugs within a time of two weeks prior to the analysis. The method described by Sheu et al. [20] was used for preparing human platelet suspensions. The platelets were suspended in Tyrode’s solution, and calcium chloride was then added, with the final concentration of Ca^2+^ being 1 mM.

The cytotoxicity of the CDOTs was evaluated using an LDH release assay. Washed platelets (3.6 × 10^8^ cells/mL) were pretreated with 50-500 μM CDOTs or a solvent control (PBS; phosphate-buffered saline) for 20 min at 37 °C and then centrifuged at 5000 g for 5 min. The supernatant obtained was used for the assay. Briefly, 10 μL of the supernatant was placed on a Fuji Dri-Chem slide (LDH-PIII) (Tokyo, Japan), and the absorbance was measured at 540 nm using a UV–vis spectrophotometer (UV-160; Shimadzu, Japan). The LDH activity of 1% Triton X-100-treated washed platelets indicated 100% LDH release.

### 2.5. Platelet Aggregation

Platelet aggregation was monitored using a lumi-aggregometer (Helena Laboratories, Beaumont, TX, USA), as previously described [20]. The platelet suspension (3.6 × 10^8^ cells/mL) was preincubated with various CDOT concentrations (25–120 μM) or an isovolumetric solvent control (PBS) for 3 min before the agonists were added. The reaction was permitted to continue for at least 10 min and the level of aggregation was calculated in light transmission units. The amplitude and slope of platelet aggregation were automatically calculated using the aggregometer.

### 2.6. Western Blotting

Washed platelets (1.2 × 10^9^ cells/mL) were preincubated with the CDOTs (65 μM and 90 μM) for 3 min before treating them with collagen to induce platelet activation. A lysis buffer (200 μL) was used for platelet resuspension after the reaction was complete. Proteins (80 μg) from the supernatants were separated using 12% SDS-PAGE and electrophoretically transferred to PVDF membranes (Bio-Rad, Hercules, CA, USA). The membranes were blocked with 5% BSA in Tris-buffered saline (10 mM Tris-base, 100 mM NaCl, and 0.01% Tween 20) for 1 h and probed with various primary antibodies, followed by incubation with horseradish peroxidase-labeled anti-rabbit or anti-mouse immunoglobulin G antibodies for 1 h. Antibody-bound proteins on the membranes were detected using an ECL system and quantified using Bio-profil Biolight (version V2000.01; Vilber Lourmat, Marne-la-Vallée, France).

### 2.7. Tail Bleeding Time in Mice

ICR mice (20–25 g, 5–6 weeks old, male) were obtained from BioLasco (Taipei, Taiwan). All procedures and protocols were approved by the Affidavit of Approval of Animal Use Protocol, Taipei Medical University (LAC-2018-0360). The bleeding time was measured after 10 min of intravenous administration of 1 mg/kg CDOTs or PBS (control). The tail of anesthetized mice was cut 3 mm from the end and then directly immersed in normal saline at 37 °C. The bleeding time was recorded until no sign of bleeding was observed for at least 10 s.

### 2.8. ADP-Induced Acute Pulmonary Thromboembolism in Mice

According to our previous method, we used ADP to induce acute pulmonary thromboembolism in mice [21]. A fixed dose of the CDOTs (1 mg/kg) or PBS was intravenously injected, and after 10 min, ADP (0.7 mg/g) was injected into the tail vein. The lungs were then removed and placed in 4% formalin, and paraffin-embedded sections were stained with hematoxylin–eosin and then photographed using ScanScope CS (Leica Biosystems, Wetzlar, Germany). The mortality rate was recorded in all animal groups within 1 h of the injection.

### 2.9. Statistical Analysis

Data are expressed as mean ± standard error of the mean (SEM), and convoyed by the number of observations (*n*). n represents the number of experiments, and each experiment was performed using different blood donors. Statistical significances were analyzed for the in vivo experiments using unpaired Student’s *t* test. One-way analysis of variance (ANOVA) was implemented to determine variations between the experimental groups and, if the analysis exhibited a significant difference, they were compared using the Student–Newman–Keuls test. *p* < 0.05 indicated statistical significance.

## 3. Results

### 3.1. Characterization of the CDOTs

#### 3.1.1. X-ray Diffraction Analysis

**Figure 1:**
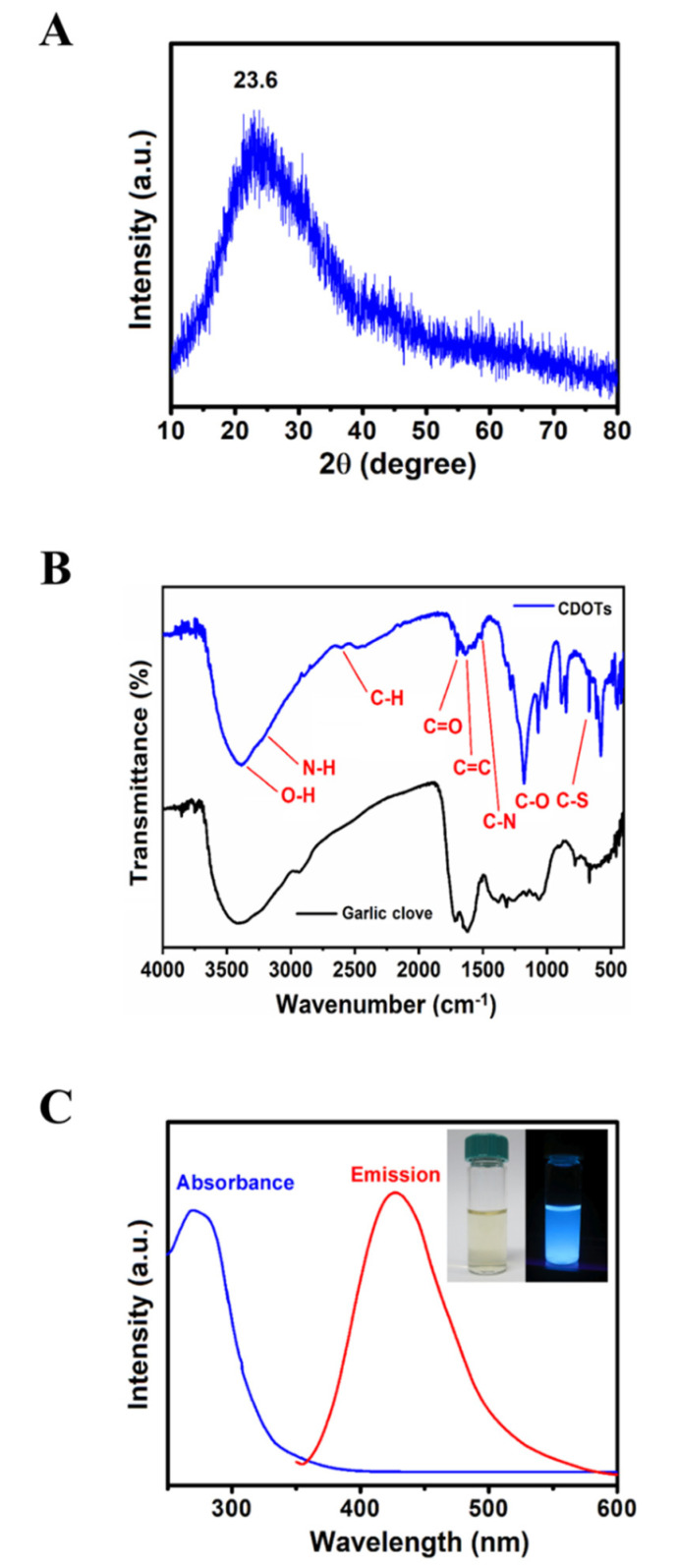
Characterization of the synthesized CDOTs. (**A**) X-ray diffraction (XRD), (**B**) Fourier transform infrared (FT–IR) spectra, and (**C**) UV–vis absorption spectra, as described in the Materials and Methods section.

The X-ray diffraction (XRD) pattern revealed that one diffraction peak at 2θ of 23.6° corresponded to disordered carbon atoms and the (002) graphite lattice, as shown in Figure 1A, and this finding was consistent with that previously reported for CDOTs [22].

#### 3.1.2. FT–IR

The FT–IR spectrum observed for the garlic clove is rather similar to that of the CDOTs, indicating that the functional groups were, indeed, successfully provided the garlic clove, as illustrated in Figure 1B. The broad absorption band centering at 3427 cm^−1^ should be associated with the O–H stretching vibration mode of the hydroxyl functional groups in the garlic clove. The weak bands at 2940 and 1413 cm^−1^ confirm the presence of CH_2_ groups, whereas the bands at 921 and 1568 cm^−1^ revealed the presence of oxygen-containing functional groups. The peaks at approximately 2944 and 1405 cm^−1^ were assigned to the C–H and C–N stretching vibration modes, and the absorption at 680 cm^−1^ could be ascribed to the C–S group [23]. Consequently, the as-prepared CDOTs were mainly composed of different functional groups on their surface, which is favorable for sustainable applications in biology.

#### 3.1.3. UV–vis Spectroscopy

The UV–vis absorption spectra of the CDOTs, as shown by the blue line in Figure 1C, showed a comparable absorption band ranging from 200 to 600 nm, concordant with an earlier study on N-doped CDOTs produced by Wu et al. [24]. The CDOTs water solution produced solid blue light under UV irradiation of 365 nm, as shown by the right inset in Figure 1C. The CDOTs exhibited very strong FL in the range of 380–600 nm, with the maximum peak at around 446 nm, as shown by the red line in Figure 1C.

### 3.2. LDH Assay and FE-TEM

**Figure 2:**
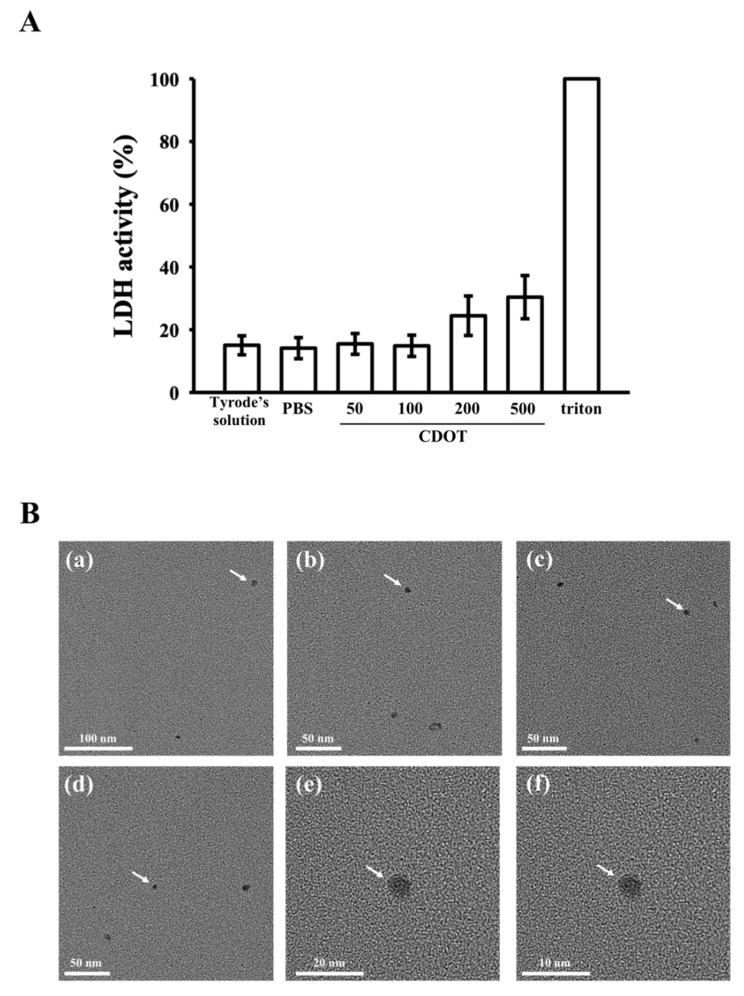
Cytotoxicity and morphology of the CDOTs. (**A**) Washed platelets (3.6 × 10^8^ cells/mL) were preincubated with PBS (control) or the synthesized CDOTs (50, 100, 200 and 500 μM) for 20 min, and a 10 μL suspension of the supernatant was deposited on a Fuji Dri-Chem slide (LDH-PIII). (**Ba–f**) Field-emission transmission electron microscopic images. The arrows indicate sizes and morphologies of CDOTs. Values represent mean ± SEM (*n* = 6).

Herein, we explored the probable toxic effects of the synthesized CDOTs on platelets by observing the release of cytosolic LDH. The CDOTs (50 µM and 100 µM) did not provoke any substantial discharge of LDH from platelet cytosol, even at concentrations of up to 200 µM, as shown in Figure 2A. Thus, they evidently did not disturb platelet membrane integrity or induce cytotoxicity at concentrations as high as 200 µM. A slight increase was observed at a higher concentration of 500 µM. LDH activities measured from the 1% Triton X-100-treated platelets were regarded as 100% release.

The morphological features and average particle sizes of the CDOTs are shown in Figure 2B. The synthesized CDOTs had a crystalline structure and were well distributed in water without aggregation. Furthermore, they were round in shape with a normal diameter of 3 nm [25].

### 3.3. Inhibition of Platelet Aggregation Stimulated by Collagen

**Figure 3:**
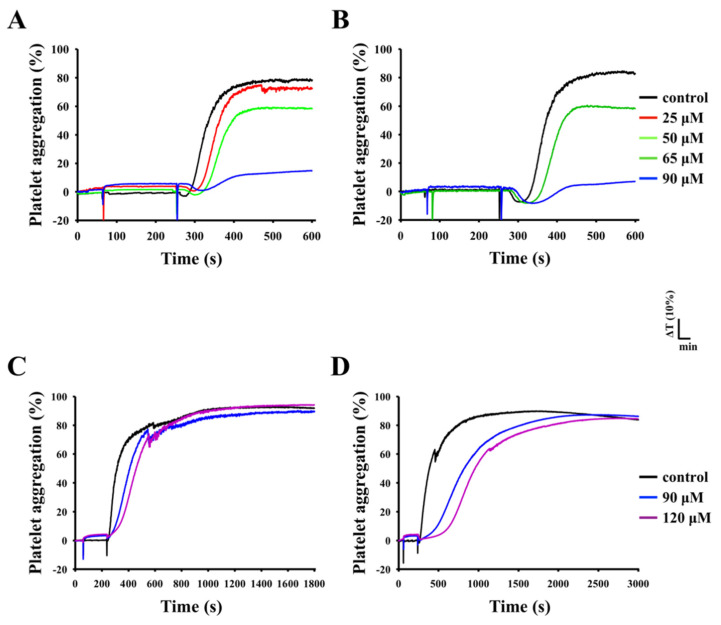
Inhibitory effects of the CDOTs on human platelet aggregation. Washed platelets (3.6 × 10^8^ cells/mL) were preincubated with PBS (control) or the synthesized CDOTs (25-120 μM) and subsequently treated with (**A**,**B**) 1 μg/mL collagen, (**C**) 1 μM U46619, and (**D**) 0.01 U/mL thrombin to induce platelet aggregation. The IC50 and maximal inhibitory concentrations are shown in **B**. The inhibitory profiles (**A**–**D**) are representative examples of four similar experiments. The delayed lag phase of platelet aggregation noticed in CDOT-pretreated platelets stimulated by either U46619 (**C**) or thrombin (**D**).

**Figure 4:**
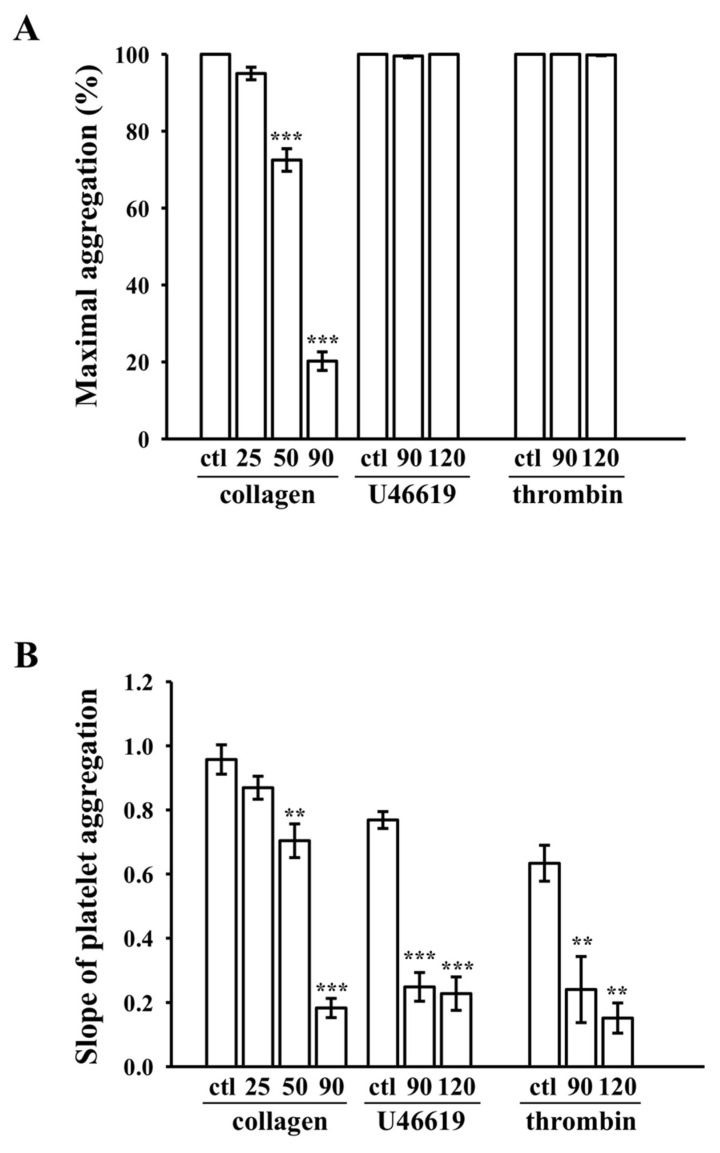
Maximal aggregation and slope of aggregation curves. (**A**) Concentration-response bar diagrams of the synthesized CDOTs, demonstrating their inhibitory activity for maximal aggregation (%). (**B**) Slope of platelet aggregation, as calculated from the linear part of the aggregation trace. Values represent mean ± SEM (*n* = 4). ** *p* < 0.01 and *** *p* < 0.001, compared with the control (ctl; PBS-treated) group.

The CDOTs led to concentration-dependent (25–120 µM) inhibition of platelet aggregation induced by collagen (1 µg/mL), as shown in Figure 3A,B, but not by U46619 (1 µM), a prostaglandin endoperoxide (thromboxane A_2_ receptor agonist), or thrombin (0.01 U/mL), even at higher concentrations of 120 µM, as shown in Figure 3C,D. Almost full inhibition was observed at 90 µM in collagen stimulated aggregation, as shown in Figure 3B. As a result, the IC_50_ (65 µM) and maximal concentration (90 µM) of the CDOTs were chosen to observe the potential inhibitory mechanisms in collagen-activated platelets. The CDOTs suppressed maximal platelet aggregation, stimulated by collagen, but not by U46619 and thrombin, as shown in Figure 4A, whereas the slopes of platelet aggregation revealed that the CDOTs also significantly reduced the lag time induced by these agonists, respectively, as shown in Figure 4B.

### 3.4. Inhibition of PKC Activation (p47; Pleckstrin) and Akt, JNK1/2, and p38 MAPK Phosphorylation

**Figure 5:**
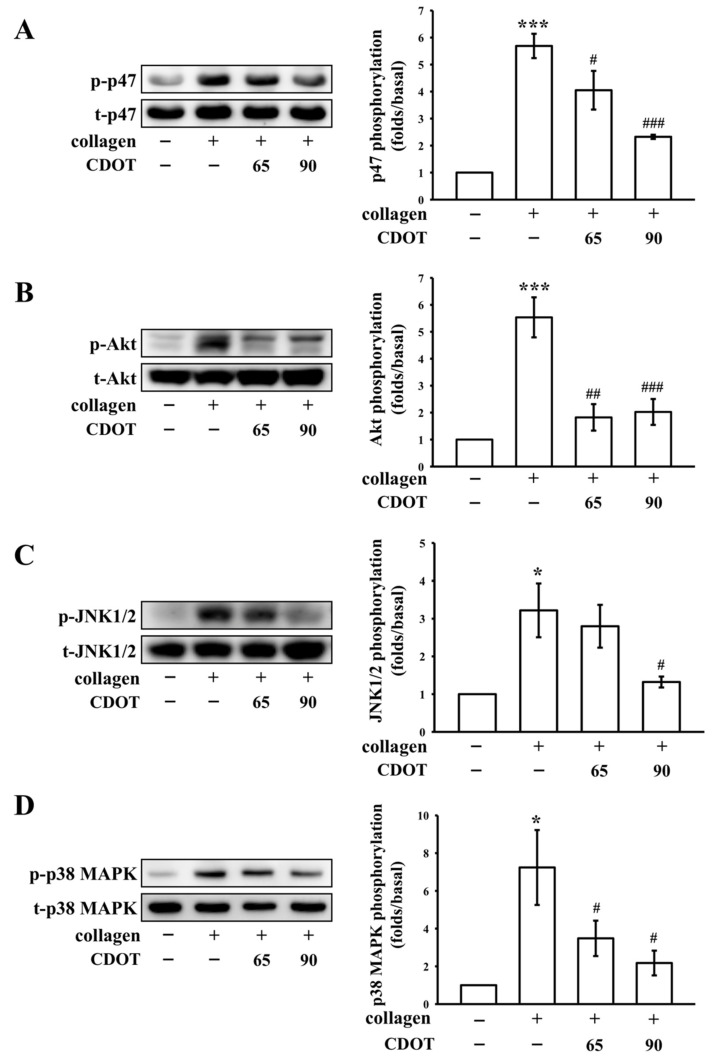
Effects of the CDOTs on PKC activation, and Akt, JNK1/2, and p38 MAPK phosphorylation in collagen-activated platelets. Washed platelets (1.2 × 10^9^ cells/mL) were preincubated with PBS (control) or the synthesized CDOTs (65 and 90 μM), and subsequently, collagen (1 μg/mL) was added to trigger (**A**) PKC activation (p-p47) and (**B**) Akt, (**C**) JNK1/2, and (**D**) p38 MAPK phosphorylation. Values represent mean ± SEM (*n* = 4). * *p* < 0.05 and *** *p* < 0.001, compared with the control (PBS-treated) group. # *p* < 0.05, ## *p* < 0.01, and ### *p* < 0.001, compared with the collagen-treated group.

We additionally investigated the mechanisms by which the CDOTs inhibited platelet aggregation. Their effects on PKC activation (p-p47) and Akt (protein kinase B), JNK1/2, and p38 MAPK phosphorylation are shown in Figure 5. The CDOTs (65 and 90 µM) significantly and, in a concentration-dependent manner, suppressed PKC activation in collagen-activated platelets, as shown in Figure 5A. Akt is a serine/threonine-specific protein kinase, which acts a major element in numerous cellular events, such as platelet activation, cell proliferation, apoptosis, and cell migration [26]. The CDOTs markedly inhibited collagen-induced Akt phosphorylation, as shown in Figure 5B. Furthermore, the CDOTs inhibited JNK1/2 and p38 MAPK [27] phosphorylation, which were elevated in collagen-stimulated platelets, as shown in Figure 5C,D, respectively.

### 3.5. Effects of the CDOTs on Tail Bleeding and Mortality in Mice with ADP-Induced Pulmonary Thromboembolism

**Figure 6:**
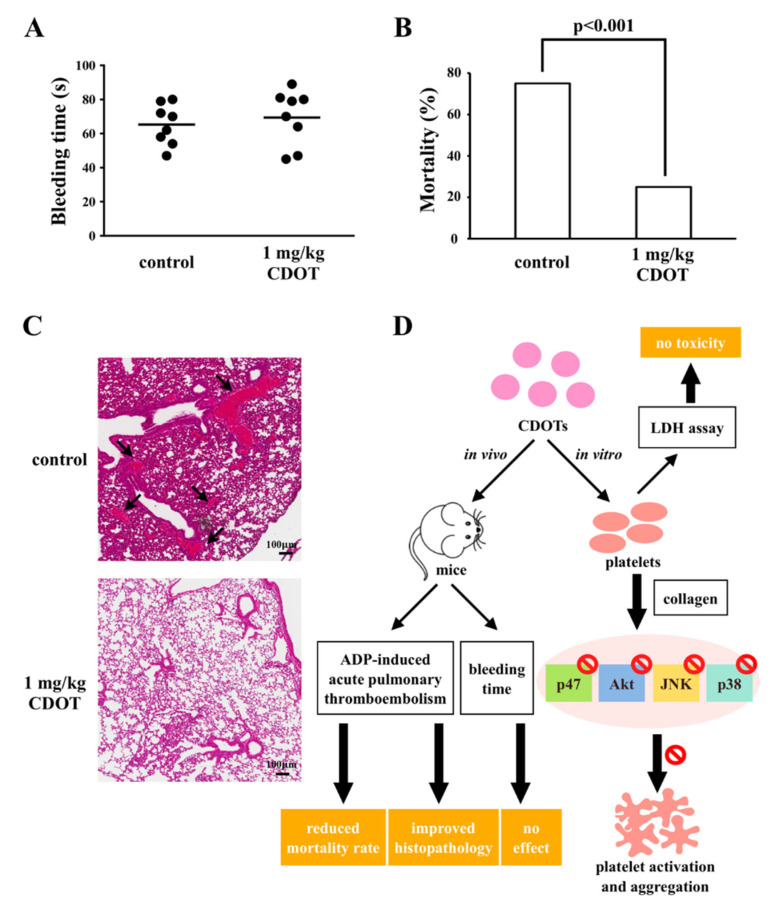
Effects of CDOTs on tail bleeding time and pulmonary thrombosis in experimental mice. (**A**) Bleeding time was measured through tail transection after 10 min of intravenous administration of PBS (control) or 1 mg/kg CDOTs. The bleeding time was continuously recorded until no sign of bleeding was observed for at least 10 s. (**B**) For the study of acute pulmonary thrombosis, PBS (control) or 1 mg/kg CDOTs was intravenously administered to mice, and ADP (0.7 mg/g) was then injected through the tail veins. (**C**) Pulmonary thrombosis (arrows) was observed by staining lung tissue sections with hematoxylin–eosin. Scale bar: 100 μm. Values represent mean ± SEM (*n* = 8). (**D**) Schematic illustration showing the inhibitory effect of CDOTs in human platelets. CDOTs potently inhibit human platelet activation by suppressing PKC activation and Akt, JNK1/2, and p38 MAPK phosphorylation without inducing cytotoxicity. CDOTs reduced the mortality in ADP-induced thromboembolic mice and did not affect bleeding tendency.

Bleeding is a common side-effect of the antiplatelet drugs used in this study. We evaluated the effects of the CDOTs on bleeding time via a tail transection model. The bleeding time was 65.3 ± 4.2 s (*n* = 8) in the control group, as shown in Figure 6A. After 10 min of intravenous administration of the CDOTs (1 mg/kg), the bleeding time was 69.4 ± 5.7 s (*n* = 8). As is evident, the bleeding time was not significantly affected.

Further, we investigated mortality in mice with ADP-induced acute pulmonary thromboembolism treated with the CDOTs. The mortality rate of the animals with ADP-induced acute pulmonary thromboembolism (0.7 mg/g ADP) was 75% (i.e., deaths of 6 mice, *n* = 8); however, pretreatment with the CDOTs (1 mg/kg) considerably reduced the mortality rate to only around 25% (i.e., deaths of 2 mice, *n* = 8), as shown in Figure 6B. Hematoxylin–eosin was used to stain the lung tissues of the mice. As shown in Figure 6C, the control group exhibited severe pulmonary thrombosis (arrows), whereas the CDOTs (1 mg/kg) exerted substantial protective effects. Overall, these results showed that the synthesized CDOTs had an eminent antiplatelet effect in vivo without the side-effect of bleeding.

## 4. Discussion

CDOTs have extensively been applied in different fields for drug delivery. They have also been used in bioimaging and as effective biosensors for protein detection [14], considering their excellent biocompatibility, good water solubility, low toxicity, high photoluminescence, and high photostability. In this study, we synthesized a new type of CDOT from garlic. These nanoparticles were potent at hindering collagen-induced platelet aggregation and only reduced the slope of the aggregation curve (lag time) by U46619 and thrombin. Different physiological agonists (e.g., collagen, thrombin, and ADP) activated platelets. The primary activation of agonists may be enriched by the secondary activation induced by thromboxane A_2_ from arachidonic acid or by ADP from the granules in platelets. In the case of blood vessel injury, platelets adhere to the subendothelial matrix (collagen), causing granule secretion and platelet activation. Collagen, a matrix protein which exists in the vascular subendothelium and vessel wall, acts as substrate for platelet adhesion and potent platelet stimulator. In this manner, collagen exerts as a key player in platelet activation.

To exclude the possible cytotoxic effects of the synthesized CDOTs on human platelets, we estimated the leakage of cytosolic LDH. LDH, a soluble cytoplasmic enzyme which occurs in nearly all cells is released into the extracellular space when the plasma membrane is injured. We found that the alteration between the control and platelets subjected with 200 µM CDOTs was not substantial, suggesting potential hemocompatibility. This result is concordant with that reported by Shrivastava et al. [28], who established that silver nanoparticles did not disturb platelet membrane integrity, even at concentrations as high as 500 μM. In addition, LDH release was not noticed from platelets after exposure to 0.9–3.5 nM silver nanoparticles [29]. Moreover, in a recent study, Hajtuch et al. [30] reported that functionalized silver nanoparticles, such as AgNPs-GSH, AgNPs-PEG, and AgNPs-LA, ranging in size from 2 to 3.7 nm, inhibited platelet aggregation without releasing LDH. The results pertaining to the effects of nanoparticles on platelets are inconsistent. Studies have found that gold nanoparticles are inert [31] or activate [32] platelets. Silver nanoparticles have been reported to induce platelet aggregation both in human platelets and in an animal models [33]. Huang et al. [34] demonstrated that silver nanoparticles coated with polyvinyl pyrrolidone and citrate had no significant effects on human platelet aggregation. In this study, we found that the synthesized CDOTs effectively inhibited collagen-triggered platelet aggregation. Consistent with our findings, Ragaseema et al. [35] reported the inhibitory effects of silver nanoparticles on platelet aggregation. In addition, Shrivastava et al. [28] found that silver nanoparticles condensed ADP- and collagen-induced platelet activation with a reduction in the slope of aggregation. These inconsistencies could be attributed to differences in size, stabilization, and functionalization, as well as the method of nanoparticle synthesis.

In the present study, the CDOTs evidently inhibited collagen-stimulated platelet activation, implying that they were effective in inhibiting platelet activation via a prominent phospholipase C (PLC)-dependent mechanism. PLC, belonging to a family of kinases, hydrolyzes phosphatidylinositol 4,5-bisphosphate to yield two chief secondary messengers: diacylglycerol and inositol trisphosphate. Diacylglycerol activates PKC-inducing pleckstrin (p47) phosphorylation and ATP release in activated platelets, whereas inositol trisphosphate elevates calcium influx [36]. The observed antiplatelet effects of the CDOTs could be a result of the inhibition of the PLC–PKC cascade, leading to the suppression of Akt and MAPK activation. Akt (a downstream regulator of PI3K)-knockout mice have been found to demonstrate impaired platelet activation [22]. Hence, Akt inhibition may be considered as striking antithrombotic targets. Conservative MAPKs are classified into ERK1/2, p38 MAPK, JNK1/2, and big MAPK (ERK5) [37]. ERK1/2, JNK1/2, and p38 MAPK participate in platelet activation [37]. MAPK presents in platelets linked to the mechanistic role of several antiplatelet agents [38]. Adam et al. [39] reported that JNK1 knockdown reduced platelet aggregation, with JNK1^−/−^ platelets displaying abnormal platelet granule secretion, and led to defective thrombus formation in mice. p38 MAPK is associated with thrombus formation, as evidenced in p38 MAPK^−/−^ mice [39,40]. Therefore, PKC, Akt, JNK1/2, and p38 MAPK are regarded as major targets of antiplatelet agents. A study demonstrated that silica nanoparticles induced expressions of the phosphorylated JNK and p38 MAPK, and suppressed ERK phosphorylation in human umbilical vein endothelial cells [41]. In the current study, the synthesized CDOTs markedly inhibited collagen-induced PKC, Akt, JNK1/2, and p38 MAPK phosphorylation in a concentration-dependent manner.

The GPVI receptor induces strong signaling through the protein tyrosine kinase pathways that results in the activation of PI3 and PLCγ and Ca^2+^ release. Since, in this study, CDOT effectively inhibited collagen-induced platelet aggregation, GPVI receptor-mediated inhibitory signaling pathways maybe involved in this anti-aggregatory effect. Thus, we believe that the inhibition of these signaling molecules by the CDOTs may lead to inhibitory effects on platelet activation. Miller et al. proved the hypothesis that the biological activity of nanoparticles may be dictated by their composition, size, and charge [42]. They found that human- or bovine-derived nanoparticles inhibited platelet aggregation induced by two different agonists—one that activates the thrombin receptor and the other that activates the collagen receptor—and they suggested that the inhibitory effects may be nonspecific, possibly by reducing platelet–platelet interactions or by binding to these or other surface receptors. Consistent with these discoveries, the current in vitro observation of the potent inhibitory effect of CDOTs in collagen-induced human platelet aggregation may be due to its inhibition of platelet–platelet interactions or by preventing binding with the collagen receptor. However, the detailed mechanisms of these hypotheses remain to be explored.

The intravenous administration of nanoparticles has been previously reported to substantially inhibit platelet aggregation in mice, indicative of their in vivo antiplatelet effects [28]. Furthermore, Shrivastava et al. [28] conducted tail-bleeding assays to determine the presence of any opposing effect on bleeding time and found that mice continued to live normally after nanoparticle administration. Similarly, Kim et al. [43] found that silver nanoparticles were nontoxic to rodents, and in another more relevant study, gold nanoparticles were observed to inhibit both thrombosis and considerably improve the survival rates of mice, without increasing the bleeding risk [44]. These results are consistent with those of this study, where even we found that CDOT administration reduced mortality in thromboembolic mice without prolonging the bleeding tendency.

## 5. Conclusions

CDOTs have recently gained much attention worldwide. Herein we found that the synthesized CDOTs could actively inhibit human platelet activation by suppressing PKC activation and Akt, JNK1/2, and p38 MAPK phosphorylation. Furthermore, there was no cytotoxicity in vitro. The in vivo study revealed that the CDOTs had an antithrombotic effect on the ADP-induced pulmonary thromboembolic mice model. CDOTs attenuate ADP-induced severe pulmonary thrombosis via the potential recovering of lung histopathology, reducing mortality and maintaining the normal bleeding tendency in mice. Altogether, our results suggest that a direct application of CDOTs may contribute to the development of new antiplatelet drugs for the treatment of arterial thromboembolic diseases.

## References

[1] Watson R.D., Chin B.S., Lip G.Y. (2002). Antithrombotic therapy in acute coronary syndromes. Br. Med. J..

[2] Baruah D.B., Dash R.N., Chaudhari M.R., Kadam S.S. (2006). Plasminogen activators: A comparison. Vasc. Pharmacol..

[3] Almoosa K. (2002). Is Thrombolytic therapy effective for pulmonary embolism. Am. Fam. Physician.

[4] Maxwell M.J., Yuan Y., Anderson K.E., Hibbs M.L., Salem H.H., Jackson S.P. (2004). SHIP1 and Lyn Kinase negatively regulate integrin αIIbβ3 signaling in platelets. J. Biol. Chem..

[5] Scuri M., Chen B.T., Castranova V., Reynolds J.S., Johnson V.J., Samsell L., Walton C., Piedmonte G. (2010). Effects of titanium dioxide nanoparticle exposure on neuroimmuneresponses in rat airways. J. Toxicol. Environ. Health A.

[6] Buffle J. (2006). The key role of environmental colloids/nanoparticles for the sustainability of life. Environ. Chem..

[7] Hasan A., Morshed M., Memic A., Hassan S., Webster T.J., Marei H.E. (2018). Nanoparticles in tissue engineering: Applications, challenges and prospects. Int. J. Nanomed..

[8] Berry J.P., Arnoux B., Stanislas G., Galle P., Chretien J. (1977). A microanalytic study of particles transport across the alveoli: Role of blood platelets. Biomedicine.

[9] Gaffney A.M., Santos-Martinez M.J., Satti A., Major T.C., Wynne K.J., Gun’ko Y.K., Annich G.M., Elia G., Radomski M.W. (2015). Blood biocompatibility of surface-bound multi-walled carbon nanotubes. Nanomedicine.

[10] Anselmo A.C., Modery-Pawlowski C.L., Menegatti S., Kumar S., Vogus D.R., Tian L.L., Chen M., Squires T.M., Sen Gupta A., Mitragotri S. (2014). Platelet-like nanoparticles: Mimicking shape, flexibility, and surface biology of platelets to target vascular injuries. ACS Nano.

[11] Roy S.C., Paulose M., Grimes C.A. (2007). The effect of TiO2 nanotubes in the enhancement of blood clotting for the control of hemorrhage. Biomaterials.

[12] Gayen B., Palchoudhury S., Chowdhury J. (2019). Carbon dots: A mystic star in the world of nanoscience. J. Nanomater..

[13] Lim S.Y., Shen W., Gao Z. (2015). Carbon quantum dots and their applications. Chem. Soc. Rev..

[14] Zuo J., Jiang T., Zhao X., Xiong X., Xiao S., Zhu Z. (2015). Preparation and application of fluorescent carbon dots. J. Nanomater..

[15] Wang R., Lu K.-Q., Tang Z.-R., Xu Y.-J. (2017). Recent progress in carbon quantum dots: Synthesis, properties and applications in photocatalysis. J. Mater. Chem. A.

[16] Yan X., Zhao Y., Luo J., Xiong W., Liu X., Cheng J., Wang Y., Zhang M., Qu H. (2017). Hemostatic bioactivity of novel Pollen Typhae Carbonisata-derived carbon quantum dots. J. Nanobiotechnol..

[17] Zhao Y., Zhang Y., Liu X., Kong H., Wang Y., Qin G., Cao P., Song X., Yan X., Wang Q. (2017). Novel carbon quantum dots from egg yolk oil and their haemostatic effects. Sci. Rep..

[18] Liu X., Wang Y., Yan X., Zhang M., Zhang Y., Cheng J., Lu F., Qu H., Wang Q., Zhao Y. (2018). Novel phellodendri cortex (huang bo)-derived carbon dots and their hemostatic effect. Nanomedicine.

[19] Fedel M. (2020). Hemocompatibility of carbon nanostructures. C J. Carbon Res..

[20] Sheu J.R., Lee C.R., Lin C.H., Hsiao G., Ko W.C., Chen Y.C., Yen M.H. (2000). Mechanisms involved in the antiplatelet activity of Staphylococcus aureus lipoteichoic acid in human platelets. Thromb. Haemost..

[21] Lu W.J., Lee J.J., Chou D.S., Jayakumar T., Fong T.H., Hsiao G., Sheu J.R. (2011). A novel role of andrographolide, an NF-κB inhibitor, on inhibition of platelet activation: The pivotal mechanisms of endothelial nitric oxide synthase/cyclic GMP. J. Mol. Med..

[22] Zhu C., Zhai J., Dong S. (2012). Bifunctional fluorescent carbon nanodots: Green synthesis via soy milk and application as metal-free electrocatalysts for oxygen reduction. Chem. Commun. (Camb.).

[23] Alkian I., Prasetio A., Anggara L., Karnaji, Fonisyah M.H., Rizka Z.M., Widiyandari H. (2019). A facile microwave-assisted synthesis of carbon dots and their application as sensitizers in nanocrystalline TiO_2_ solar cells. J. Phys. Conf. Ser..

[24] Wu Z.L., Zhang P., Gao M.X., Liu C.F., Wang W., Leng F., Huang C.Z. (2013). One-pot hydrothermal synthesis of highly luminescent nitrogen-doped amphoteric carbon dots for bioimaging from bombyx mori silk—Natural proteins. J. Mater. Chem. B.

[25] Zhao S., Lan M., Zhu X., Xue H., Ng T.W., Meng X., Lee C.S., Wang P., Zhang W. (2015). Green synthesis of bifunctional fluorescent carbon dots from garlic for cellular imaging and free radical scavenging. ACS Appl. Mater. Interfaces.

[26] Woulfe D.S. (2010). Akt signaling in platelet and thrombosis. Expert Rev. Hematol..

[27] Bugaud F., Nadal-Wollbold F., Levy-Toledano S., Rosa J.P., Bryckaert M. (1999). Regulation of c-jun-NH2 terminal kinase and extracellular-signal regulated kinase in human platelets. Blood.

[28] Shrivastava S., Bera T., Singh S.K., Singh G., Ramachandrarao P., Dash D. (2009). Characterization of antiplatelet properties of silver nanoparticles. ACS Nano.

[29] Krishnaraj R.N., Berchmans S. (2013). In vitro antiplatelet activity of silver nanoparticles synthesized using the microorganism Gluconobacter roseus: An AFM-based study. RSC Adv..

[30] Hajtuch J., Hante N., Tomczyk E., Wojcik M., Radomski M.W., Santos-Martinez M.J., Inkielewicz-Stepniak I. (2019). Effects of functionalized silver nanoparticles on aggregation of human blood platelets. Int. J. Nanomed..

[31] Love S.A., Thompson J.W., Haynes C.L. (2012). Development of screening assays for nanoparticle toxicity assessment in human blood: Preliminary studies with charged Au nanoparticles. Nanomedicine.

[32] Deb S., Patra H.K., Lahiri P., Dasgupta A.K., Chakrabarti K., Chaudhuri U. (2011). Multistability in platelets and their response to gold nanoparticles. Nanomedicine.

[33] Jun E.A., Lim K.M., Kim K., Bae O.N., Noh J.Y., Chung K.H., Chung J.H. (2011). Silver nanoparticles enhance thrombus formation through increased platelet aggregation and procoagulant activity. Nanotoxicology.

[34] Huang H., Lai W., Cui M., Liang L., Lin Y., Fang Q., Liu Y., Xie L. (2016). An evaluation of blood compatibility of silver nanoparticles. Sci. Rep..

[35] Ragaseema V.M., Unnikrishnan S., Kalliyana Krishnan V., Krishnan L.K. (2012). The antithrombotic and antimicrobial properties of PEG-protected silver nanoparticle coated surfaces. Biomaterials.

[36] Singer W.D., Brown H.A., Sternweis P.C. (1997). Regulation of eukaryotic phosphatidylinositol-specific phospholipase C and phospholipase D. Ann. Rev. Biochem..

[37] Fan X., Wang C., Shi P., Gao W., Gu J., Geng Y., Yang W., Wu N., Wang Y., Xu Y. (2018). Platelet MEKK3 regulates arterial thrombosis and myocardial infarct expansion in mice. Blood Adv..

[38] Mazharian A., Roger S., Berrou E., Adam F., Kauskot A., Nurden P., Jandrot-Perrus M., Bryckaert M. (2007). Protease-activating receptor-4 induces full platelet spreading on a fibrinogen matrix: Involvement of ERK2 and p38 and Ca2+ mobilization. J. Biol. Chem..

[39] Adam F., Kauskot A., Nurden P., Sulpice E., Hoylaerts M.F., Davis R.J., Rosa J.P., Bryckaert M. (2010). Platelet JNK1 is involved in secretion and thrombus formation. Blood.

[40] Adam F., Kauskot A., Rosa J.P., Bryckaert M. (2008). Mitogen-activated protein kinases in hemostasis and thrombosis. J. Thromb. Haemost..

[41] Guo C., Xia Y., Niu P., Jiang L., Duan J., Yu Y., Zhou X., Li Y., Sun Z. (2015). Silica nanoparticles induce oxidative stress, inflammation, and endothelial dysfunction in vitro via activation of the mapk/nrf2 pathway and nuclear factor-kappab signaling. Int. J. Nanomed..

[42] Miller V.M., Hunter L.W., Chu K., Kaul V., Squillace P.D., Lieske J.C., Jayachandran M. (2009). Biologic nanoparticles and platelet reactivity. Nanomedicine.

[43] Kim Y.S., Kim J.S., Cho H.S., Rha D.S., Kim J.M., Park J.D., Choi B.S., Lim R., Chang H.K., Chung Y.H. (2008). Twenty-eight-day oral toxicity, genotoxicity, and gender-related tissue distribution of silver nanoparticles in Sprague-Dawley rats. Inhal. Toxicol..

[44] Tian Y., Zhao Y., Zheng W., Zhang W., Jiang X. (2014). Antithrombotic functions of small molecule-capped gold nanoparticles. Nanoscale.

